# The clinical relevance of progestogens in hormonal contraception: Present status and future developments

**DOI:** 10.18632/oncotarget.26015

**Published:** 2018-10-02

**Authors:** Pedro-Antonio Regidor

**Affiliations:** ^1^ Exeltis Europe & Germany, 85737 Ismaning, Germany

**Keywords:** progestogen, contraception, pharmakokinetik

## Abstract

The contraceptive pill is an effective and very safe method to control pregnancies. It was developed 60 years ago, and despite, that the composition has been the same since it was first developed (estrogen and progestogen), along the years the concentration of ethinyl estradiol has been reduced to improve tolerability. Nevertheless, progestogens are the basic active agent of hormonal contraception. The mechanism of progestogens is a multimodal one and basically three modes of contraceptive action can be distinguished:

(a) A strong antigonadotrophic action leading to the inhibition of ovulation. The necessary dosage of ovulation inhibition per day is a fixed dosage that is inherent to each progestogen and independent of the dosage of estrogen used or the partial activities of the progestogen or the mode of application. (b) Thickening of the cervical mucus to inhibit sperm penetration and (c) Desynchronization of the endometrial changes necessary for implantation.

The on the market available progestogens used for contraception are either used in combined hormonal contraceptives (in tablets, patches, or vaginal rings) or as progestogen only contraceptives. Progestogen only contraceptives are available as daily oral preparations, monthly injections, implants (2-3 years), and Intrauterine Systems (IUS). Even the long acting progestogens are highly effective in typical use and have a very low risk profile, with few contraindications.

According to their introduction into the market progestogens, in combined hormonal contraceptives, have been described as first, second, third and fourth generation progestogens. Also, progestogens can be derived from testosterone, progesterone, and spironolactone that determine pharmacodynamic and pharmacokinetic differential effects. These effects contribute to the tolerability and additional beneficial or therapeutic effects whether used in combined oral contraceptives COC or as progestogen only drugs enhancing the individual options for different patient profiles.

The new development of polymers for vaginal rings allowed on one side the improvement of the estrogen/progestogen combination in these rings especially regarding the comfort of use for women (avoiding of cold chain use or packages with up to six-month rings e.g.) and on the other side the development of progestogen only formulations. Another future development will be the introduction of new progestogen only pills that will provide effective contraceptive protection with more favourable bleeding patterns and a maintenance of ovulation inhibition after scheduled 24-h delays in pill intake than the existing pop with desogestrel.

## INTRODUCTION

Since starting the use of oral hormonal contraceptives as combined estrogen/progestogen drugs in 1960, combined oral contraceptives (COC) have experienced a continuous change in used progestogens showing changing aspects of the partial effects in addition to its inhibition of ovulation [1]. These aspects of progestogen actions have been shown to be useful in incorporating aspects of non-contraceptive use into the possibilities of therapeutic uses creating a wide range of positive effects besides the primary use as contraceptives, such as protection for endometrial and ovarian cancer, dysmenorrhea and endometriosis symptoms relief and decreasing in menstrual flow. During the last decades also changes in regard of the used estrogens have been implemented [1]. A general awareness to the non-contraceptive benefits of hormonal contraceptives must be reached, as these drugs, besides their objective high efficacy and safety have been shown to have non-contraceptive benefits. In addition to their clinical value in different medical indications, COC’s have a very favorable cost/benefit ratio and a good level of compliance in comparison to other drugs used for medical indications. It has been stated that the non-contraceptive health benefits of COC’s represent an important aspect of the overall impact of this group of drugs beyond their primary use [2].

It is also important to point out that:

Thirteen percent of women aged between 15 and 19 years become pregnant voluntarily or not each year, a ratio that has not changed statistically since the 70s [3].

Approximately eighty-five percent of the above-mentioned pregnancies are unintended [4]. The economic and social impact factors of the one million teenage pregnancies each year in USA represent an important political factor [4].

Avoiding unintended pregnancies is a main concern of most sexually active women, especially the adolescents. It is estimated that in the year 1995 eighty-one percent of the women aged between 15 and 19 years at risk of unintended pregnancy were using contraceptive methods; many of them reported using two methods—one to avoid sexual transmitted diseases (STDs) and the second as the contraceptive one. The most used contraceptive methods were either oral contraceptives (44% of the cases), male condoms (46% of the cases) or in 8% both systems. However, the success with these methods depends heavily on user compliance. Especially with adolescents the problems are the incorrect intake of the tablets or failure to use condom just before sexual intercourse. These are USA data, but this topic is similar in almost all countries of the world [5].

These data emphasize the need of an effective contraception. The newer progestogens dienogest and drospirenone in combination with estrogens are therefore useful tools in the management of both; effective contraception to avoid unintended pregnancies and by the same way to offer non-contraceptive health benefits. Table 1 depicts the partial effect patterns of these two progestogens and of others.

**Table 1:** Chemical structure of progestogens and the year of introduction into the market

Structural Groups		Progestogen	Year of synthetization and/or market introduction
Progesterone		Progesterone	1933/1997
Retroprogesterone		Dydrogesterone	1959
Progesterone Derivate		Medrogestone	1964
17a-Hydroxyprogesterone Derivates	Pregnanes (C21)	Medroxyprogesterone acetate	1957
		Megestrol	1959
		Chlormadinone acetate	1959
		Cyproteroneacetate	1961
17a-Hydroxynorprogesterone Derivates	Norpregnanes (C20)	Nomegestrol acetate	1986
		Gestenoron Caproate	1973
19-Norprogesterone Derivates	Norpregnanes (C19)	Demegestone	1974
		Promegestone	1983
		Nestorone	2001
		Trimegestone	2001
19-Nortestosterone Derivates	Estranes (C18)	Norethindrone	1951
		Norethisterone acetate	1951
		Lynestrenol	1961
		Norethinodrel	1957
		Ethynodiol Acetat	1967
19-Nortestosterone Derivates	Gonanes (C17)	Norgestrel	1966
		Levonorgestrel	1966
		Desogestrel	1981
		Etonogestrel	1998
		Gestoden	1986
		Norgestimate	1986
		Dienogest	1978
Spirolonactone Derivate		Drospirenone	1976

By the same way every prescription should also take into consideration the potential risks associated with the use of COC; e.g. the occurrence of thromboembolic events.

These risks remain very low and are under continuous evaluation. The last referrals of the European Medicine Agency of 2014 [6] rated the risk for the use of COC with drospirenone at 9-12 cases under 10.000 users (0,1 %) and declared 2017 [7] that this risk for dienogest containing COC with ethynyl estradiol is still unknown and may be lower than that of other progestogens in combined formulations.

Nevertheless, new developments in contraception as e.g. estrogen free contraceptive systems intend to reduce these cardiovascular side effects by maintaining a high efficacy. Here the introduction of a new drospirenone only pill can be a milestone.

## HISTORICAL DEVELOPMENT OF PROGESTOGENS

In 1951 C. Djerassi and L. Miramontes converted 3-methoxy-estradiol into a 19-nortestosterone derivate with the help of the Birch reduction. In the next steps this 19-nortestosterone derivate was subsequently transformed by means of several chemical steps into 17α-ethinyl-19- nortestosterone (norethisterone) [8]. Norethisterone progestogenic potency was about 20-fold higher than that of ethisterone. 19-norprogesterone was also synthesized in 1951 by G. Rosenkranz and C. Djerassi using the same chemical method (Birch reduction). This substance was orally inactive, but it represented a potent progestogen after parenteral administration [8]. This progestogen is the basic substance of a series of 19-nor progesterone derivatives that have been applied till the beginning of this century (e.g., norhydroxyprogesterone caproate) or are still used today for contraception and/or hormone therapy as efficient progestogens (e.g., trimegestone, segesterone acetate, NOMAC).

Schering Germany developed due to the work of Junkmann and Schenk in 1951 norethisterone acetate. F. Colton synthesized norethynodrel at the G. D. Searle company from Chicago, Illinois. Afterwards dimethisterone that was developed in 1957 in England. The first use of this relatively weak progestogen was contraception, especially in sequential oral contraceptives [9].

Dimethisterone, like other progestogens, disappeared from the market. It was also Junkermann who developed 1954 at Schering the first progesterone derivative: 17α-acetoxyprogesterone. Medroxyprogesterone acetate, megestrol acetate and chlormadinone acetate followed in the years 1957, 1957 and 1959 respectively (all at Syntex). The prodrugs of norethisterone lynestrenol and ethynodioltediacetat like norethynodrel, and D, L-norgestrel were synthesized in the 1960s [9].

P. Duphar developed in 1959 the retroprogesterone derivative dydrogesterone (Philips-Duphar); in 1961 cyproterone acetate was synthesized by R. Wiechert at Schering. Desogestrel 1972 at Organon and dienogest 1978 by Hübner and Ponsold at Jenapharm [9].

Wiechert and collaborators synthesized in 1976 drospirenone at Schering AG. However, it took about 25 years, until its pharmacological potential was detected, and the drug brought into the market in the year 2000 [10]. Table 2 depicts the different progestogen groups in relation to their chemical structure and year of development.

**Table 2:** Relative binding affinities of progestogens to steroid receptors and serum binding proteins modified from [1, 13]

	PR	AR	ER	GR	MR	SHBG	CBG	Albumin Bound	Free
Progesterone	50	0	0	10		0	36	79.3	2.4
Dydrogesterone	75	0	–	–	–	–	–		
Chlormadinone acetate	67	5	0	8	0	0	0		
Cyproterone acetate	90	6	0	6	8	0	0		
Medroxyprogesterone acetate	115	5	0	29	160	0	0		
Megestrol acetate	65	5	0	30	0	0	0		
Nomegestrol	125	6	0	6	0	0	0		
Promegestone (R5020)	100	0	0	5	53	0	0		
Drospirenone	35	65	0	6	230	0	0		
Norethisterone	75	15	0	0	0	16	0	60.8	3.7
Levonorgestrel	150	45	0	1	75	50	0	50	2.5
Norgestimate	15	0	0	1	0	0	0		
Desogestrel (Etonogestrel)	150	20	0	14	0	15	0	65.5	2.5
Gestodene	90	85	0	27	290	40	0	24.1	0.6
Dienogest	5	10	0	1	0	0	0		

PR = progesterone receptor (promegestone = 100 %), AR = androgen receptor (metribolone = 100 %), ER = estrogen receptor (estradiol-17β = 100 %), GR = glucocorticoid receptor (dexamethason = 100 %), MR = mineralocorticoid receptor (aldosterone = 100 %), SHBG = sex hormone binding globulin (dihydrotestosterone = 100 %), CBG = corticosteroid-binding globulin (cortisol = 100 %).

## BIOLOGICAL ACTIVITIES OF PROGESTOGENS

Because of the enormous variation in the chemical structure of steroids with a progestogen activity, it is difficult to deduct various biological actions and activities from the chemical structure alone [11]. One of the essential requirements of any compound with such an activity is of course: being able to bind to the progesterone receptor and thus some knowledge has been developed about the three-dimensional structure required for a steroid to bind. Adding to the confusion is the fact that there appears to be several different forms of the progesterone receptors usually called PR-A and PR-B, the difference being a sequence of amino-acids in the B form that is not found in the A form. The b form can be considered as the agonistic receptor in many organs and is able to antagonize the effects stimulated by an activated a form [12, 13].

## DEVELOPMENT AND CLASSIFICATION OF PROGESTOGENS IN CONTRACEPTION

The progestogens used for contraception, like all major steroid hormones are characterized biochemically by a C 21 carbon skeleton. Different progestogens have been developed, from the basic C21 skeleton for contraception. Ethinyl substitution of testosterone was found to result in an orally active compound (ethisterone). Removal of the carbon at the C-19 position of ethisterone changed ethisterone from an androgen to a progestin, resulting in the development of a class of progestins referred to as 19-nortestosterone derivatives. Included in this class are commonly used progestins such as norethindrone, norethindrone acetate, levonorgestrel and ethynodiol diacetate.

The first orally active progestogens used in combined hormonal contraceptives were norethynodrel and norethisterone [1, 9].

The reason for the development of other progestogens was the observation that the early progestogens had androgenic properties leading to unwanted effects on lipids and the skin. The progestogens used in Combined Hormonal Contraceptives can be classified according to different criteria as follows:

## THE TIME OF INTRODUCTION INTO THE MARKET

It has become common use to apply this “historical” classification. According to this 1st, 2nd, 3rd and 4th generation combined hormonal contraceptives can be distinguished:

1st generation: Norethynodrel, Norethisteron Acetate, (NET, NETA).

2nd generation: Levonorgestrel (LNG).

3rd generation: Gestodene, Desogestrel, Norgestimate (GEST, DES, NGM).

4th generation: Drospirenone (DROSP).

Cyproterone Acetate (CPA) and Chlormadinone Acetate (CMA) have never been included in this categorization as CPA containing oral contraceptives were originally classified as drugs to treat hyperandrogenism in women who required contraception. CMA was only introduced in some countries and was and is not internationally available. The same was true for Dienogest (DNG) which was developed in Germany and is mainly used in still Germany [14].

## CLASSIFICATION ACCORDING TO MOLECULAR STRUCTURE

The molecular structure gives an indirect indication about the biologic action of the steroid. Different groups of progestogens can be distinguished [14]. These are described in Table 1.

## CLASSIFICATION ACCORDING TO INTERACTION WITH STEROID RECEPTORS

Depending on the structure progestogens have different interactions with the various steroid receptors in the body. Steroid receptors are located on the membrane of target cells and are linked to the DNA/RNA and Protein production via different messenger systems.

### The receptors to which progestogens bind can be the following:

Progestogen receptor: The most important receptor to induce the desired effect.

Androgen receptor: Activation of androgen receptors mediate androgenic effects on hair growth and activity of the sebaceous glands. Some progestogens bind to this receptor and can either block or activate it (antiandrogenic properties see below).

Estrogen receptor: This receptor mediates effects in many tissues especially in the endometrial cells.

Glucocorticoid receptor: The glucocorticoid effect is linked to the activation of the coagulation system.

Mineralocorticoid receptor: This receptor mediates sodium retention.

Based on this classification of receptor activities several groups of progestogens can be differentiated.

Androgenic, antiandrogenic, mildly antiandrogenic or neutral and antimineralocorticoid progestogens: Only Drospirenone has an antimineralocorticoid action.

The different binding affinities are shown in Table 2 and the different receptor effects are described in Table 3 [1].

**Table 3:** Contraceptive progestogens and extra-progestogenic effects (adapted from Regidor and Schindler [14])

	Progestogen	Anti-gonadotrop	Anti-estrogen	Estrogen	Androgen	Anti-androgen	Gluco-corticoid	Anti-mineralo corticoid	Pro Coagulatory
**Progesterone**	+	+	+	−	−	+/−	+	+	−
**Dydrogesterone**	+	−	+	−	−	+/−	−	+/−	−
**Medrogestone**	+	+	+	−	−	+/−	−	−	
**17a-Hydroxy-Progesterone Derivates**									
**Chlormadinonacetate**	+	+	+	−	−	+	+	−	−
**Cyproteronacetate**	+	+	+	−	−	++	+	−	−
**Megestrolacetate**	+	+	+	−	+/−	+	+	−	+
**Medroxyprogesteroneacetate**	+	+	+	−	+/−	−	+	−	
**19-Nor-Progesteron-Derivates**									
**Nomegestrolacetate**	+	+	+	−	−	+/−	−	−	−
**Promegeston**	+	+	+	−	−	−	−	−	−
**Trimegeston**	+	+	+	−	−	+/−	−	+/−	−
**19-Nortestosterone-Derivates**									
**Norethisterone**	+	+	+	+	+	−	−	−	+
**Lynestrenol**	+	+	+	+	+	−	−	−	−
**Norethinodrel**	+/−	+	+	+	+	−	−	−	−
**Levonorgestrel**	+	+	+	−	+	−	−	−	−
**Norgestimate**	+	+	+	−	+	−	−	−	−
**Desogestrel**	+	+	+	−	+	−	−	−	−
**Gestoden**	+	+	+	−	+	−	+	+	−
**Dienogest**	+	+	+/−	+/−	−	+	−	−	−
**Spirolonactone Derivate**									
**Drospirenone**	+	+	+	−	−	+	−	+	−

Classification regarding the release form

The existing pharmaceutical forms are classified in two categories:

a) Immediate release formulations

b) Controlled release formulations (or drug delivery systems)

Oral systems are a combination of immediate release formulations and controlled release. The drug plasma levels are not intended to be found more then 24 hours after application. Skin patches, vaginal delivery systems and subcutaneous implants are controlled release formulations with drug plasma levels of 7 days in the case of the patch, up to 21 days in the case of vaginal rings, between 3 months and 5 years in the case of 3-month depot formulations or intrauterine devices and 5 years for the subcutaneous implants.

## PROGESTOGEN IMPLANTS

Hormonal implants are subdermal inserted contraceptives that provide reliable contraception for 3–5 years. The matrices are inert or biologically degradable rods or capsules which release the respective steroid continuously over a period. The Population Council in New York has studied long-term contraception with subdermal hormonal implants since 1966. The hormone implants consist of one or several small flexible rods or a capsule inserted under the skin of the upper arm. Depending on the product, they release the progestins megestrol acetate, norethindrone, norgestrinone or etonogestrel for a period of 1 year to 5 years.

Norplant^®^ was composed of six rods. Each rod contains 36 mg of levonorgestrel. The total duration of action of these 6 rods was 5 years. The product has not been marketed since 2002.

Norplant II^®^ (Jadelle^®^), Norplant^®^’s successor product, is composed of two flexible silicone rods (43 mm a–2.5 mm) each containing 75 mg of levonorgestrel and has a duration of action of 5 years. The same product is commercially available in China under the name Sinoplant^®^ [15].

## THE ETONOGESTREL RELEASING HORMONAL IMPLANT IMPLANON^®^

Composition: Implanon^®^ is an etonogestrel-releasing hormonal implant. The rod is 4 cm long and 2 mm in diameter and is composed of 40 % ethylene vinyl acetate (EVA) and 60 % (68 mg) etonogestrel (3-keto-desogestrel). The duration of action after subdermal implantation is 3 years.

Mechanism of Action: The main effect is ovulation inhibition, although this inhibitory effect is less at the end of the 3 years. Additional contraceptive effects are the change in the composition of the cervical mucus and making the endometrium less receptive for a theoretical implantation. The PI is 0.38 pregnancies per 100 women-years of use, which is like that of other long-acting methods of contraception.

The concentration falls over time at a rate that depends on body weight. Clinical experience with Implanon^®^ in women weighing more than 80 kg is limited. Available data do not show a decrease in efficacy in obese women.

Accurate placement is crucial to the product’s reliability. There are reports of incorrect insertion of the Implanon^®^ rod, possibly making the contraceptive rod impossible to palpate and difficult to find. To make the product easier to use safely and simpler to locate, the system was upgraded with Implanon NXT^®^. Efficacy may be hampered by drugs affecting the metabolism of etonogestrel like antiviral drugs. No major health risks are known. There is no concern regarding bone loss. Contraindications include Breast Cancer, active liver disease, benign and malignant liver tumors (except nodular hyperplasia).

Side Effects: As with other progesterone only contraceptives, the most frequent side effect are bleeding disorders. These lead to various degrees of discontinuation, (approximately 15–18 % in USA and Europe and 3–4 % in Southeast Asia and Russia. In approximately 5 % of users the following side effects were reported: acne, headache, weight gain, mastalgia, vaginal infections and bleeding disorders. Interactions with broad-spectrum antibiotics, St. John’s wort, several antiepileptic agents and mood-altering drugs have been documented. It should be borne in mind that placement and removal require special training.

Additional Benefits: Clinical studies have shown that Implanon^®^ is also effective in treating heavy dysmenorrhea. However, the product is not approved for that purpose. Its use in this therapeutic indication is an off-label use Another possible beneficial effect is the diminution of pain caused by endometriosis [15].

## INJECTIONS (INTRAMUSCULAR AND SUBCUTANEOUS)

Medroxy progesterone acetate (DMPA) is available for injection as a depot either. In a dose of: 150 mg/1 mL injected intramuscularly or in a dose of 104 mg/0.65 mL injected subcutaneously. Both preparations last for 3 months. The mechanism of action of DMPA is like other progestogens and includes a strong antigonadotrophic effect, inhibition of ovulation, inhibition of endometrial proliferation, and changes in the cervical mucus making the mucus impenetrable for sperm. The dose is a standard dose, and no adjustment is necessary for body weight. There is no reduction in efficacy with concurrent medication.

## HEALTH RISKS AND BENEFITS

No major health risks have been found. However, long term DMPA use induces an unwanted increase of LDL Cholesterol and reduces peripheral arterial flow-mediated dilatation, which are matters of concern. DMPA does not alter coagulation factors nor increase blood pressure. Some studies have found an increased VTE risk, but these studies have methodological weaknesses and a small number of cases. Nonetheless the World Health Organization has attributed category 23 to DMPA in women with current VTE, a history of stroke or ischemic heart disease.

The primary concern is the effect of DMPA on bone density. DMPA suppresses endogenous estrogen production from the ovaries by its strong antigonadotrophic action. Compared to nonusers, the bone mineral density at the hip and spine of DMPA users decreases by 0.5–3.5 % after 1 year and 5.7–7.5 % after 2 years of use. There is therefore concerns about the use of DMPA in adolescents when the accumulation of bone mass is at its peak, and in premenopausal women as there may be an increased rate of bone density loss. However, many studies have shown that the bone loss is reversible and the best evidence available at present indicates that that DMPA does not reduce peak bone mass and does not increase the risk of osteoporotic fractures in later life in women with an average risk of osteoporosis [16, 17, 18, 19].

At present, DMPA is contraindicated when pregnancy is planned within the next year, in the presence of osteoporosis and known risk factors for fractures, or in the presence of hypothalamic amenorrhea, anorexia nervosa or chronic glucocorticoid therapy.

Side effects include menstrual irregularities (during the first 3–6 months irregular bleeding and spotting, later there may be amenorrhea in up to 75 % of users). There may be weight gain of between 3 and 6 kg (especially in young obese women). Headache, abdominal discomfort and pain, dizziness, nervousness, and asthenia have also been described.

The benefits include, reduction of heavy menstrual bleeding due to the high incidence of amenorrhea after longer use, a reduced risk of pelvic inflammatory disease, reduction of endometriotic pain, fewer painful crisis in women with sickle cell disease and reduction in vasomotor symptoms [20].

## LEVONORGESTREL CONTAINING INTRAUTERINE SYSTEMS

There are two types of Levonorgestrel containing intrauterine systems (a) Levonorgestrel-releasing intra-uterine device IUD (LNG 20)—the LNG20 IUD consists of a T-shaped polyethylene frame with a collar containing 52 mg of levonorgestrel dispersed in polydimethylsiloxane attached to a vertical stem. *In vivo*, the LNG 20 IUD initially releases 20 mcg of levonorgestrel daily, declining to 10–14 mcg per day after 5 years. The LNG 20 IUD should be replaced after 5 years (b) Levonorgestrel-releasing IUD (LNG 14) It contains 13.5 mg levonorgestrel, which is initially released at a rate of 14 mcg daily, declining to 5 mcg/day after 3 years. It is smaller in size (T shape 28 by 30 mm) than the LNg20 (T shape 32 by 32 mm) and has a smaller inserter diameter (3.8 mm versus 4.75 mm). It contains a silver ring to distinguish it on ultrasound from other IUDs and make it detectable by X-ray. The LNg14 IUD should be replaced after 3 years.

Both types of Levonorgestrel-releasing IUD’s act by thickening the cervical mucus, making it impervious to sperm, by causing endometrial decidualization and glandular atrophy, inducing expression of glycodelin A in endometrial glands which inhibits the binding of sperm to the ovum and in partial inhibition of ovulation (in approx. 20 % of users).

Both types of Levonorgestrel-releasing IUDs are efficacious. The cumulative pregnancy rate is 0.5–1.1 after 5 years of continuous use with the LNG 20 IUD. The 3-year cumulative pregnancy rate is 0.9 with the LNG 14 IUD.

Contraindications include severe deformity of the uterine cavity, acute sexually transmitted infections, unexplained vaginal bleeding, current breast cancer, Wilson’s disease and known or suspected pregnancy.

The major side effect is irregular bleeding, which is very common during the first 3–6 months. At 24 months 50 % of LNG20 users have amenorrhea, 25 % have oligomenorrhea and 11 % have spotting. The pattern is similar with the LNG 14 IUD with less amenorrhea (13 versus 24 % after 3 years).

Other side effects are rare and include breast tenderness, mood changes and acne.

Recent data of Morch et al. [21] postulate that levonorgestrel increases the risk of breast cancer. This increase was not only observed under the use of oral progestin only formulations with LNG (adjusted relative risk of 1.93, 95% CI 1.18 to 3.16) but also upon the users of LNG IUD’s (RR 1.21; 95% CI 1.11 to1.33).

There are many benefits to using the Levonorgestrel-releasing IUD. The major benefit is reduction in heavy menstrual bleeding and dysmenorrhea in patients without organic pathology and bleeding due to bleeding diathesis including anticoagulation therapy. The efficacy regarding reduction of bleeding intensity in women with fibroids and adenomyosis is yet unclear and under investigation. The Levonorgestrel-releasing IUD protects from pelvic inflammatory disease, due to cervical mucus thickening which acts as a barrier towards ascending infections. The Levonorgestrel-releasing IUD can be used to treat endometriosis. There is endometrial protection in premenopausal and menopausal women using estrogen replacement, and a concomitant reduction of the risk of endometrial cancer. The Levonorgestrel-releasing IUD can also be used to treat endometrial hyperplasia and cancer and in some cases of early stage well differentiated endometroid cancer, as an alternative approach in women who desires fertility preservation.

LNG IUDs and especially the LNG14 IUD are a highly effective method of contraception in women under age 20 due to the facilitation of compliance and the high continuation rate. Rates of infection are similar in adolescent users to those of adults. Nulliparity is not a contraindication for the use of this method of contraception [22, 23].

## PROGESTIN ONLY PILL (=POP)

The basis for progestogen only contraception lies in the specific action of progestogens on reproductive physiology. These actions are type and dose dependent and include:

Inhibition of ovulation.

Change of the cervical mucus to make it impenetrable.

Endometrial changes which make implantation either difficult or impossible.

Changes in tubal mobility.

The common clinical important features of these methods are:

Progestogens have very little impact on the coagulation system (see above). Their effects on blood flow and contractility of vessel walls is very limited. Epidemiological studies do not show any significant risk for thromboembolic venous or arterial disease. Therefore, progestogen only contraceptives can be used in women who have a contraindication for combined hormonal contraceptives (WHO MEC Category 4) or where the use is not advised (Category 3) [24]. The main contraindications to combined hormonal contraception include:

Postpartum (during the first 21 days, lactating women), combination of cardiovascular risk factors (obesity, smoking, age), venous thromboembolic risk factors (thrombophilia, family history, longer immobilization, acute deep venous thrombosis with anticoagulant therapy), specific arterial risk factors (hypertension, migraine with aura, valvular heart disease, past ischemic heart disease).

On the other hand, there are few contraindications to progestogen only contraception, but these include: Breast Cancer, active liver disease, benign and malignant liver tumors (except nodular hyperplasia).

The most frequent side effects attributed to the action of progestogens are acne, mild hirsutism, depressive mood, sexual pain, and weight gain. This is however not based on prospective clinical comparative trials but mainly observational data. The most frequent side effect of continuous use of progestogens is irregular bleeding.

Progestogen only contraception confers several important additional benefits:

Lactation: The progestogen only contraceptives can be used in lactating women because there is no reduction in milk production and no negative effect on the newborn.

Menstrual symptoms: Contraceptive progestogens can, due to their antimitotic and transformational action on the endometrial cells reduce the frequency and intensity of uterine bleeding. The contraceptive progestogens with ovulation inhibition can reduce dysmenorrhea. Additionally, progestogens block the synthesis of prostaglandins in the endometrium by reducing the endometrial thickness.

Menstrual Migraine: Progestogens in continuous use reduce the intensity of menstrual migraine.

Endometriosis: Progestogens can reduce the proliferative activity of the endometrium.

## OLDER ORAL PREPARATIONS

Levonorgestrel and Norethisterone only progestins are unable to inhibit ovulation, but their contraceptive effect is based on the action on the cervical mucus which becomes impenetrable to sperm. An additional effect of these progestogen only pills is effect on the endometrium desynchronising ovulation and endometrium transformation and preparation for implantation.

These preparations should be taken at the same time every day to exert the contraceptive effect. The typical user Pearl Index is between 6 and 8.

## ESTROGEN FREE PILL

The newer progestogen only pill with 75 μg of desogestrel daily is taken continuously without a 7-day break. 75 μg of desogestrel inhibits ovulation inhibition and is as effective as combined hormonal contraceptives. This pill is also called an estrogen free inhibitor of ovulation. No major health risks are known, and the pearl index is similar to the one of COC.

Breast Cancer, active liver disease, benign and malignant liver tumors (except nodular hyperplasia) are the classical contraindication.

Due to the daily intake needed for ovulation suppression there is no phase of progestogen withdrawal which the reason for the bleeding is occurring during the pill free interval during the use of combined hormonal contraceptives in the 21/7 regimen. Irregular bleeding is therefore the main complaint which may lead to discontinuation. Other progestogenic side effects such as acne, weight gain, depressed mood is rare.

In addition, there is alleviation of menstrual migraine, pain reduction in patients with endometriosis and reduction of hypermenorrhea and dysmenorrhea [25, 26, 27].

## PROGESTERONE VAGINAL RING PROGERING^®^

In some Latin American countries, a vaginal ring containing progesterone is market for contraception in women that are performing breastfeeding. The ring can be used up to 3 months and it releases approximately 10 mg of progesterone per day. The contraceptive efficacy is only proven during the breast-feeding period. The efficacy in clinical studies was more than 98.5 %. The ring causes an amenorrhea and no serious adverse effects have been recorded [28].

## EMERGENCY CONTRACEPTION

Levonorgestrel 1,5 mg as a single dose or Ulipristal acetate (UPA) 30 mg (Ella One^®^) also as a single dose are the two progestogene emergency contraceptives on the market. LNG acts by interfering with ovulation. It affects follicular development after selection of the dominant follicle but before the beginning of the pre-ovulatory rise in LH. Once the LH rise begins. LNG fails to inhibit ovulation [29]. LNG also influences muscular contractility of the fallopian tubes and concentrations of glycodelin-A inhibiting the sperm binding ability to thze zona pellucida [29, 30]. UPA is a selective progesterone receptor modulator with a direct inhibitory effect on follicular rupture that allows it to be effective even when given shortly before ovulation. The window of effectiveness is of 5 days in comparison of the 3 days of LNG [31]. The last EMA statement of 2014 acknowledge to both progestogens the efficacy in emergency contraception and a loss of this efficacy with increasing BMI [32]. If used as early as possible and in the first 3 days no difference could be observed also by Glasier et al [33].

## NEW DEVELOPMENTS AND FUTURE PERSPECTIVES

Two new innovations have been seen in the last year. First a new combined vaginal ring with ethinyestradiol and desogestrel has been launched into the market in 2017 and second the investigations ongoing regarding a new pop containing drospirenone 4mg in a 24/4 application form.

### Ornibel^®^

Ornibel^®^ is a new contraceptive vaginal ring with different polymers than the existing contraceptive ring. These new polymers allow a more stable and gradual release of both hormones just avoiding undesirable burst effects of ethynylestradiol and etonorgestrel. As the composition of the ring (core containing evatane 28% and skin containing evatane 9%) is different not only a more gradual and constant release because both drugs, but especially etonorgestrel, are in an under-saturation concentration, but also an avoiding of a cold chain for the storage of the ring is possible. This improves the feasibility of the ring making Ornibel^®^ an evolution in ring technology [34].

### Drospirenone only pill

Studies have been published describing the efficacy and special advantages of a new progestin only pill containing drospirenone.

In a prospective, multicenter study, noncomparative study conducted at 41 European sites in healthy women by Archer et al [35]. 4 mg of drospirenone (DRSP) daily for 24 days followed by a placebo for 4 days was performed. A total of 713 participants with 7638 cycles were analyzed. The overall pearl-index was 0,51. The proportion of participants with any bleeding decreased from 72% in cycle 1 to 40 % in cycle 6 and 32.1 5 in cycle 13. The overall bleeding profile improved considerably. Duijkers el al [36] could also show that despite the 4-day hormone free period and multiple intentional 24-hours delays in tablet intake, ovulation inhibition was maintained. This property distinguishes this new generation estrogen free pill from traditional POPs by allowing a “safety window” or flexibility in intake of 24 hours; that means doubling the window of intake.

And, as also known from other progestogens it also could be shown that this formulation improves safety as no change in hemostatic parameters could be observed after the intake of this new contraceptive. So DRSP did not increase the thromboembolic risk adding herby another benefit [37].

## CONCLUSIONS

Progestogens play an essential role in contraception independent if used with or without estrogens.

Even though they are different in structure and in the action profile they exhibit a multifocal mode of action in contraception. Besides the common progestogenic effect, each progestogen has a partial effect pattern, which has utmost relevance when clinically used. Effects and possible side effects can be influenced or determined by this.

This manuscript reviewed the existing possibilities in the use of different progestogens for contraception and the future development of these substances in the field of contraception.
